# Reduction of low-density lipoprotein receptor-related protein (LRP1) in hippocampal neurons does not proportionately reduce, or otherwise alter, amyloid deposition in APPswe/PS1dE9 transgenic mice

**DOI:** 10.1186/alzrt110

**Published:** 2012-04-26

**Authors:** Guilian Xu, Cameron C Green, Susan E Fromholt, David R Borchelt

**Affiliations:** 1 Department of Neuroscience, SantaFe HealthCare Alzheimer's Disease Center, Center for Translational Research in Neurodegenerative Disease, McKnight Brain Institute, University of Florida, Gainesville, FL 32610, USA; 2 Department of Educational Psychology and Learning Systems, College of Education, Florida State University, Tallahassee, FL 32306, USA

## Abstract

**Introduction:**

The low-density lipoprotein receptor-related protein (LRP1) and its family members have been implicated in the pathogenesis of Alzheimer's disease. Multiple susceptibility factors converge to metabolic pathways that involve LRP1, including modulation of the processing of amyloid precursor protein (APP) and the clearance of Aβ peptide.

**Methods:**

We used the Cre-lox system to lower LRP1 levels in hippocampal neurons of mice that develop Alzheimer-type amyloid by crosses between mice that express Cre recombinase under the transcriptional control of the GFAP promoter, mice that harbor loxp sites in the LRP1 gene, and the APPswe/PS1dE9 transgenic model. We compared amyloid plaque numbers in APPswe/PS1dE9 mice lacking LRP1 expression in hippocampus (n = 13) to mice with normal levels of LRP1 (n = 12). Student t-test was used to test whether there were significant differences in plaque numbers and amyloid levels between the groups. A regression model was used to fit two regression lines for these groups, and to compare the rates of Aβ accumulation.

**Results:**

Immunohistochemical analyses demonstrated efficient elimination of LRP1 expression in the CA fields and dentate gyrus of the hippocampus. Within hippocampus, we observed no effect on the severity of amyloid deposition, the rate of Aβ40/42 accumulation, or the architecture of amyloid plaques when LRP1 levels were reduced.

**Conclusions:**

Expression of LRP1 by neurons in proximity to senile amyloid plaques does not appear to play a major role in modulating the formation of these proximal deposits or in the appearance of the associated neuritic pathology.

## Introduction

Amyloid deposition is a key pathological event in Alzheimer's disease. A large body of evidence suggests that low density lipoprotein (LDL) receptor related protein (LRP1), may be a key mediator of amyloid deposition. As a member of the LDL receptor family, LRP1 is a large, multifunctional, endocytic receptor that is highly expressed in neurons (reviewed by Andersen [1]), activated astrocytes [2-4], and microglia [5]. Direct binding of LRP1 to the amyloid precursor protein (APP) has been shown to affect endoproteolytic processing of APP to increase the production of Aβ42 peptides [6], which are the major constituent of amyloid deposits [7]. LRP1 can promote Aβ production by altering the processing of APP through interactions via the Kunitz protease inhibitor (KPI) domain (isoforms APP751 or APP770) [6]. Although the non-KPI-APP isoform can weakly bind to LRP1 through cytoplasmic adaptor proteins, such as FE65 [8], APP695 processing may not be influenced as significantly by LRP1. Additionally, LRP1 has also been reported to mediate export of Aβ across the blood-brain barrier (reviewed by [9]); LRP1 in endothelial cells may bind directly to Aβ1-40 and export it across the blood-brain barrier [10]. Consistent with this notion, reducing LRP1 levels by antisense treatment reduces blood-brain barrier clearance of Aβ42 [11]. Polymorphisms in the LRP1 gene have been associated with increased risk for Alzheimer's disease [12] and LRP1 levels are significantly reduced in AD patients [13]. More recently, Sehgal and colleagues reported that an extract of *Withania somnifera *induces a rapid clearance of amyloid pathology with a co-incident induction of LRP1 expression in liver [14]. Other proteins implicated in amyloidogenesis have been identified as interacting with LRP1 [15], such as apolipoprotien E (ApoE) and α2-macroglobulin. Both of these proteins are ligands of LRP1 and have been reported to mediate the clearance of Aβ [13,16-19]. Moreover, ApoE, which is a major ligand for LRP1, modulates the distribution and character of amyloid deposition [20]. Collectively, these data suggest that LRP1 could be a crucial factor in Alzheimer's disease and the receptor characteristics of LRP1 make this protein a potential "drugable" target where influencing the binding of LRP1 to any of its ligands could be a useful therapeutic.

To date, there have been few studies that examine the role of LRP1 *in vivo *using animal models by genetic manipulation of LRP1 expression. Constitutive inactivation of LRP1 is lethal [21]. We [22] and others [23] have used mice with targeted deletion of LRP1 receptor associated protein (RAP), which regulates LRP1 maturation, as a means to reduce LRP1 levels in mouse models of Alzheimer's disease. One group has reported that homozygous deletion of RAP, which lowers LRP1 levels by 75% [24], results in increased Aβ deposition in PDAPP mice [23]. Similarly, we observed that heterozygous deletion of RAP also increased amyloid deposition, in the APPswe/PS1dE9 model; however, in our case LRP1 levels were not significantly lowered [22]. Expression of an LRP1 mini-receptor at approximately 3.7-fold over endogenous LRP1 levels in the PDAPP transgenic model was reported to have no effect on amyloid burden [25]. Collectively, these studies illustrate the inconsistent *in vivo *findings regarding LRP1 and amyloidogenesis in transgenic mice that over-express mutant APP (with or without co-expression of mutant presenilin).

To further probe the role of LRP1 in amyloidogenesis, we used Cre/Lox technology to conditionally reduce LRP1 expression in the brains of mice that co-express mutant APP (APPswe) and mutant presenilin 1 (PS1dE9). For these studies we used mice that express Cre under the control of the glial fibrillary acidic protein (GFAP) [26]. In previous studies with these animals, others have demonstrated Cre mediated recombination in both neurons and glial throughout the brain [27]. In our hands, GFAP-mediated expression of Cre produced incomplete recombination, such that the levels of LRP1 in total brain were reduced approximately 50%. However, we determined that LRP1 immunostaining in CA and granule neurons of the hippocampus was virtually eliminated. Within the hippocampus, we observed no obvious change in the level of neuritic pathology or amyloid burden by loss of LRP1.

## Materials and methods

### Animals

Mice with targeted Lox P sites introduced into intronic and promoter regions of the LRP1 gene were obtained from Joachim Herz [28,29], called LRP1 lox/lox. Transgenic mice expressing GFAP-Cre were obtained from Jackson Laboratories (Bar Harbor, ME, USA; Strain: FVB-Tg(GFAP-cre)25Mes/J; Stock Number: 004600) [26]. These animals were back-crossed to C57BL/6J mice for at least four generations to reduce the FVB strain contribution before breeding to LRP1 lox/lox mice. LRP1 lox/lox mice were in parallel bred to APPswe/PS1dE9 (line 85) mice [30] (both with C57BL/6J background) to produce mice homozygous for the LRP1 loxp locus. To generate triple transgenic mice, we mated mice transgenic for GFAP-Cre and homozygous for LRP1 loxp to mice transgenic for APPswe/PS1dE9 and homozygous for LPR loxp. FVB/NJ male and female mice were purchased from Jackson Laboratories (stock number: 001800) to produce foster mothers. Eventually, animals with the genotypes of APPswe/PS1dE9(+)/LRP1 lox/lox with/without the GFAP-Cre gene were used for the study. All of the mice were housed in standard SPF cages for 9 to 18 months before harvesting. All procedures involving animal handling and processing were approved by the University of Florida Institutional Animal Care and Use Committee and in compliance with the National Institutes of Health guidelines.

### Western blot analysis

Levels of LRP1 were assessed by Western blot, using standard methods we have previously described [31]. Aliquots of the PBS brain homogenates (100 μl) from 14- to 15-month-old mice were mixed with 10 μl of 10% SDS, centrifuged at the top speed (approximately 10,000 xg) in a bench top microfuge for 10 minutes, and then 30 μl of the supernatant was mixed with 10 μl 4x Laemmli buffer and boiled for 5 minutes. An amount of sample equal to 50 μg total protein (by BCA assay) were loaded per lane for SDS-PAGE and transferred to nitrocellulose membranes. Blots were incubated with rabbit polyclonal antibody 377 αLRP1 (1:1,000) and then reprobed with the m/h SOD1 antibody (1:3,000) to gauge protein loading [32]. The ECL signal was captured and quantified using the LAS-3000 imaging system (FUJIFILM Life Science, Tokyo, Japan).

### Tissue preparation for histology

Mice were deeply anesthetized by injection anesthetic (3% body weight of 1.2% Tribromoethanol (Avertin Sigma, St. Louis, MO, USA) before transcardial perfusion with cold phosphate buffered saline (PBS, pH7.4). The brains were then removed and cut sagittally down the midline; one hemi-brain was frozen on dry ice for biochemistry assay, the other hemi-brain was immersion fixed in 4% paraformaldehyde in PBS. After 48 hours in fixation at 4°C, brains were transferred to PBS, and then 30% sucrose in PBS for several days before cryostat sectioning at 30 μm thickness. From the first complete section closest to mid-line, 10 sections per group were stored in each well of 24-well-plates and the entire hemi-brain was sectioned. Sections were kept in anti-freeze solution (100 mM sodium acetate, 250 mM polyvinyl pyrrolidone, 40% ethylene glycol, pH6.5) at -20°C until staining.

### Immunostaining

Immunostaining of 30 μm free floating sections was performed according to a standard protocol. Sections were washed in PBS to remove the anti-freeze solution, and then blocked in PBS containing 5% normal goat serum with 0.1% Triton X-100 before incubating with antibodies against LRP1 (rabbit polyclonal 377 αLRP1 (1:1,000)), glial fibrillary acidic protein (GFAP) (mouse monoclonal, 1:1,000, Millipore, Billerica, MA, USA), neuronal antigen NeuN (mouse monoclonal, 1:1000, Millipore) or 6E10 (mouse monoclonal, 1:1,000, Covance, Princeton, NJ, USA). Secondary antibodies of goat anti-rabbit and goat anti-mouse, conjugated with Alexa 594 or Alexa 488 (Invitrogen, Carlsbad, CA, USA) were used to visualize primary antibody binding. After washing to remove unbound secondary antibody, the sections were then mounted on microscope slides and dried in air. A quick dip in 0.1% Sudan Black B solution (in 70% ethanol) for 5 to 10 minutes was used to block the auto-fluorescence of lipofuscin then the slides were washed in 70% ethanol, then water, dried and covered by ProLong Gold antifade reagent with Dapi (Invitrogen). An Olympus DSU-IX81 Spinning Disc Confocal microscope (Tokyo, Japan) was used to capture the images.

### Thioflavin S staining and silver staining of amyloid plaques

One section from each well (see above Tissue preparation for histology) was randomly selected for Thioflavin S staining according to the Guntern standard protocol [33] with small modification [34]. Fluorescent photos were taken using 5x objectives by a Canon digital camera (Tokyo, Japan). Silver impregnation staining was performed on the same 30 μm floating sections by modified Hirano's method [35,36]. Briefly, sections were washed and mounted on slides to dry as those for Thioflavin S staining, and then the steps described for silver impregnation methods of paraffin slides were followed [36].

### Amyloid plaque quantification

Four sections per animal (from 300 to 1,500 μm lateral to midline) stained with Thioflavin S were used to quantify the amyloid plaque levels. The images of the hippocampus were outlined and the numbers of amyloid plaques were manually counted by an observer blinded to genotype. The images of each hippocampus were outlined and quantified three times on three different days to reduce bias and then analyzed. The numbers of amyloid plaques in each section were averaged and then numbers of plaques from the four sections from different levels of the brain were averaged to produce the final number of amyloid plaques/section in the hippocampus of each animal.

### Aβ ELISA

The frozen hemi-brain from each transgenic animal was weighed, then homogenized by sonication in five volumes by weight in PBS with 1 × protease inhibitor cocktail (Sigma Cat.P8340). A 100 μl aliquot of this PBS brain homogenate was used for ELISA analysis. Protein aggregates in the PBS homogenate were denatured by the addition of 150 μl of 8.0 M guanidine-HCl to a final concentration of 4.8 M, and mixed at room temperature for three to four hours before storage overnight at -20°C. The denatured samples were then further diluted 200-fold in reaction buffer (PBS containing 5% BSA, 0.03% Tween-20, and 1 × protease inhibitor cocktail (Sigma)). Samples were finally prepared for application to the ELISA microplate by mixing with an equal volume of Standard Dilution Buffer provided by the manufacturer supplemented with 1 × protease inhibitor cocktail. Aliquots of these samples were assayed for both Aβ40 and Aβ42 using commercially available sandwich-ELISAs according to the manufacturer's manual (Invitrogen).

### Statistical analysis of the ELISA data

We compared the differences among the means of Aβ40 and 42 levels among the same age groups and the distribution of the levels across all ages. The comparison in the same age group between two different genotypes (GFAP-Cre positive or negative) was carried out using Excel with the two-tail Student *t*-test with both equal and unequal variance. The Aβ levels within each age group were graphed in Excel and linear regression lines were added. Excel Analysis ToolPak add-in was used for regression analysis. IBM SPSS Statistics 19 [(trial version), Somers, NY, USA] was used to test the significance between the regression curves by genotypes. A *P*-value of < 0.05 was considered statistically significant.

## Results

A strain of GFAP-Cre mice that expresses Cre early in neurogenesis was used to cross to the homozygous mice with LRP1 loxp sites with the expectation of complete recombination of flox'ed genes in neurons as well as glia [27]. The GFAP-Cre mice that were used for this study from the Jackson Laboratory were maintained in the FVB strain. To make them compatible with our strains of mice, we back-crossed them to C57BL/6J mice for four generations before crossing to LRP1 lox/lox mice, and then we continued to cross them to C57BL/6J congenic APPswe/PS1dE9 mice (line 85). Mice that were transgenic for APPswe/PS1dE9 and LRP1 loxp/loxp were mated to mice that were transgenic for GFAP-Cre and LRP1 loxp/loxp, or mice that were transgenic for all three alleles (APPswe/PS1dE9, GFAP-Cre and LRP1 loxp/loxp) were mated to mice only transgenic for LRP1 loxp/loxp. A total of 54 breeding cages were set up over the course of the study to generate the mice we ultimately examined. More than 20 of these cages failed to generate any offspring, 5 cages generated a few offspring, but the mothers failed to care for the litters and none of the offspring lived to weaning age (four weeks old). Of the animals of the desired genotype (APPswe/PS1dE9/LRP1loxp/loxp/GFAP-Cre) that were produced, almost half died suddenly in the first nine months of life (see Additional file 1 Table S1).

Because of the reduced viability of the mice of the desired genotype and the poor mothering of the C57BL/6J females, we began a foster breeding program. FVB/NJ females, (which are very good mothers), were used to foster all newborn offspring in an effort to increase the frequency of animals that survive to weaning. This foster-mothering program was successful in producing a greater number of animals of the desired genotype that survived to weaning. Surprisingly, the survival rate of the offspring that were foster-mothered was also much improved. A total of 167 offspring were produced in the foster-mothering program of which only 7 died before nine months of age and only 1 of these 7 was triple transgenic genotype (see Additional file 1 Table S1). Overall, the distribution of the mice with all four of the genotypes was much closer to Mendelian distribution (25% of each genotype expected). At present, we have no explanation for this observation, but it appears that the foster-mothering that improved survival to weaning age transferred to improved resistance to the underlying problem that caused the sudden early death after weaning. Ultimately most of the mice that were studied in this report were generated by foster-mothering.

### GFAP-Cre expression in LRP1 lox/lox mice lowers LRP1 levels in hippocampus

**Figure 1:**
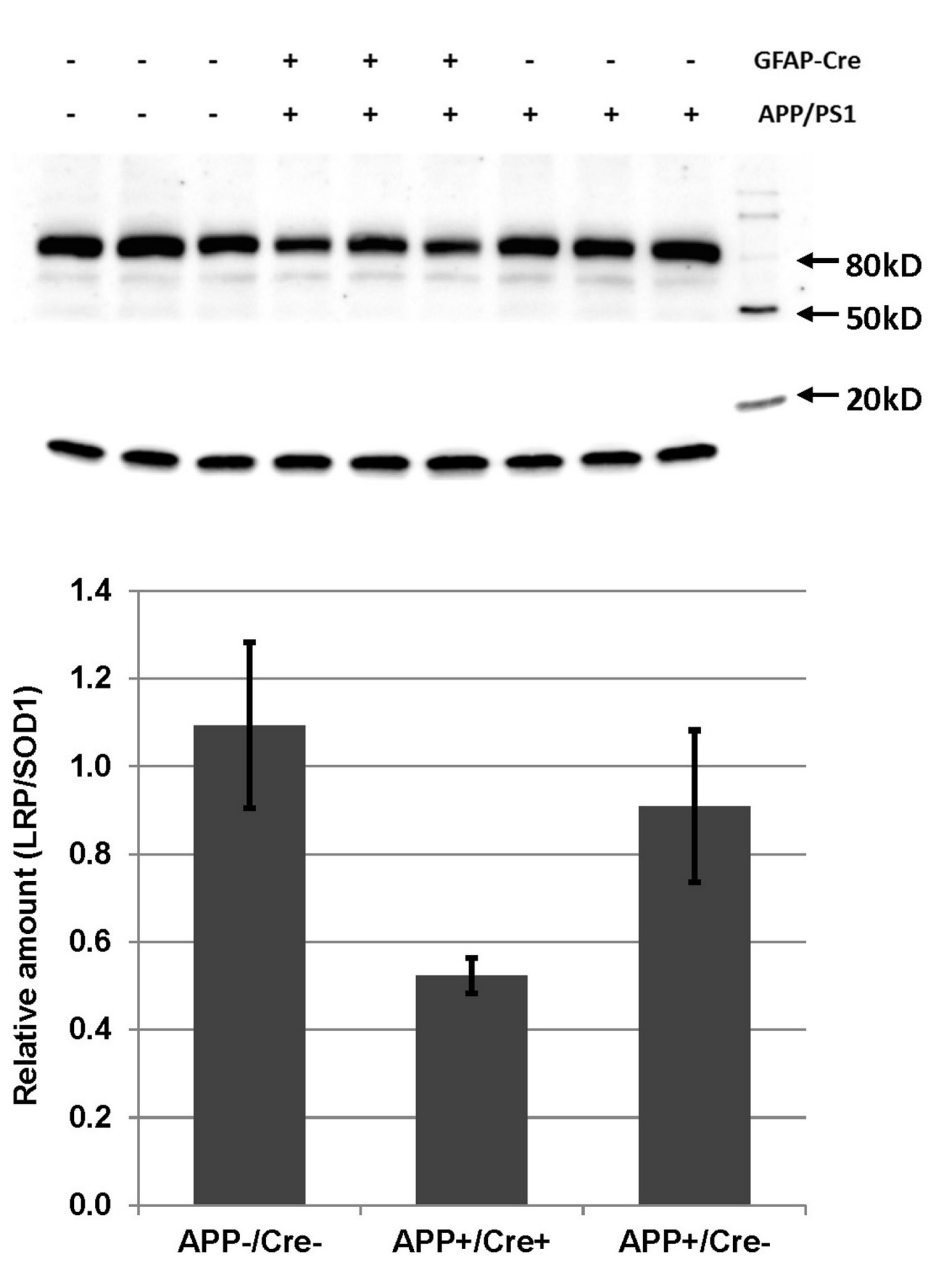
**The level of LRP1 is significantly lower in the mice expressing GFAP-Cre with LRP1 lox/lox**. Nine APPswe/PS1dE9 × GFAP-Cre × LRP1 lox/lox mice with different genotypes were used to determine the levels of LRP1. The SDS-soluble fractions of whole brain lysates (homogenized in PBS) containing an equal amount of total protein (50 μg) was loaded in each lane. Antibody α377 to LRP1 (1:1,000, a gift from Dr. Herz, University of Texas Southwestern Medical Center) was used to detect LRP1 level. The 85 kDa band is the endoproteolytically cleaved mature LRP1. Mouse SOD1 (14 kD bands) was used as endogenous loading control. The ratio of band intensity of LRP1 to SOD1 was quantified, standardized and plotted below. The standard deviations of the measurements are shown by error bars.

Using the breeding scheme described above, we produced mice transgenic for GFAP-Cre, APPswe/PS1dE9, and homozygous for the flox'ed LRP1 alleles. All mice described in this work were homozygous for the LRP1lox allele. No obvious developmental abnormalities were observed in the brains of mice that were transgenic for GFAP-Cre whether or not they were also transgenic for the additional APPswe/PS1dE9 transgenes (not shown). Immunoblots of hemi-brain homogenates demonstrated that LRP1 levels were reduced by approximately 50% in mice that were transgenic for APPswe/PS1dE9 and GFAP-Cre as compared to mice that were transgenic only for APPswe/PS1dE9 (Figure 1).

**Figure 2:**
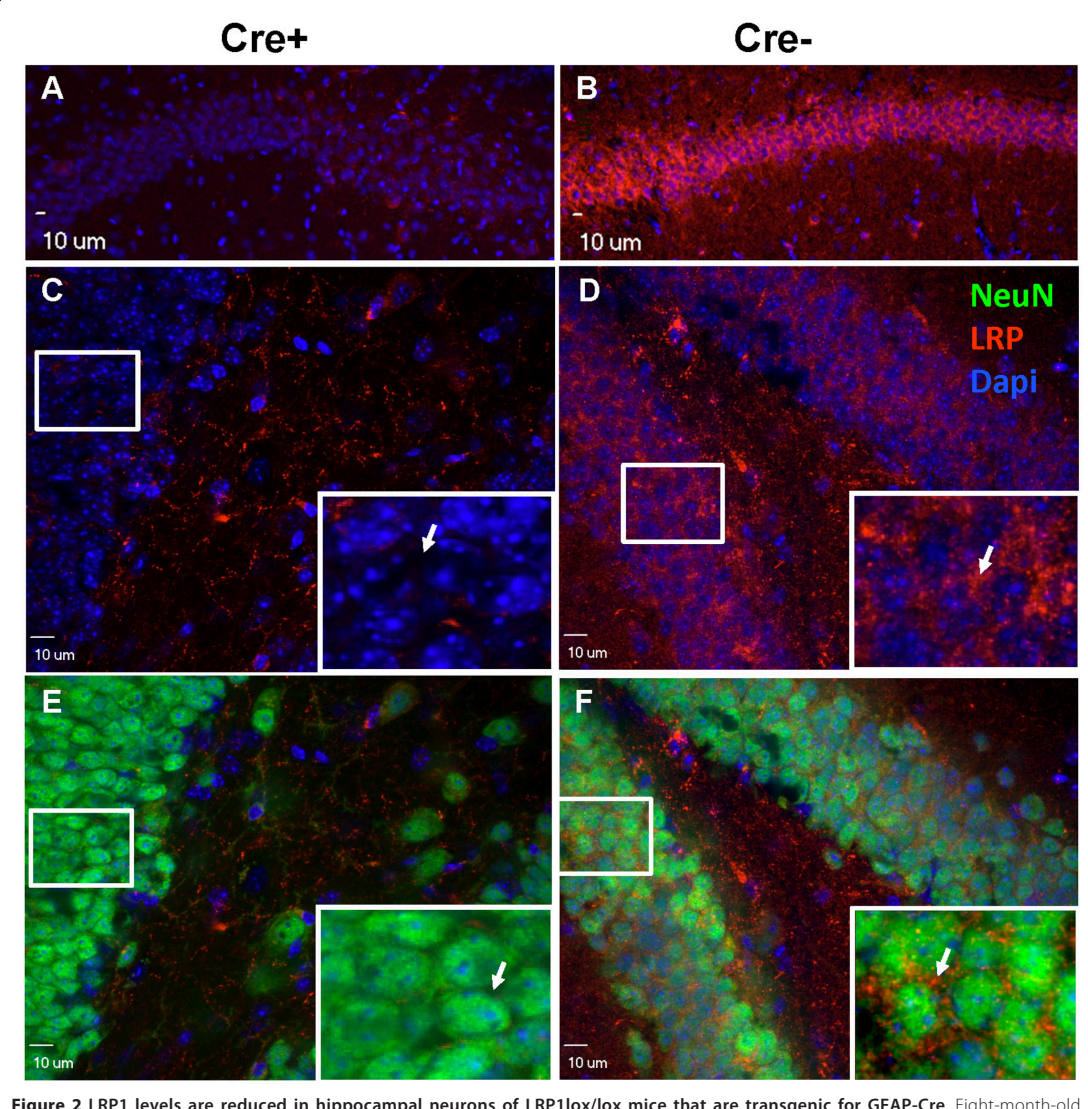
**LRP1 levels are reduced in hippocampal neurons of LRP1lox/lox mice that are transgenic for GFAP-Cre**. Eight-month-old APPswe/PS1dE9 × GFAP-Cre × LRP1 lox/lox littermates were used for immunostaining. All mice were homozygous for LRP1loxp alleles and positive for APPswe/PS1dE9 transgenes. The presence or absence of GFAP-Cre is noted above the images. LRP1 α377 antibody (1:1,000, red) was used to stain LRP1; NeuN (1:1,000, green) was used to stain neurons; Dapi in the mounting reagent was used to display nuclei. Confocal microscope images showed most of the neurons in CA fields **(A) **and the dentate gyrus **(C **and **E) **of APPswe/PS1dE9 × LRP1lox/lox × GFAP-Cre mice do not exhibit LRP1 immunoreactivity. Control mice lacking the GFAP-Cre transgene showed intense staining of LRP1 in hippocampal neurons **(B, D**, and **F)**. Three to four pairs of mice per genotype were used for immunostaining and four to five sections per animal were analyzed.

**Figure 3:**
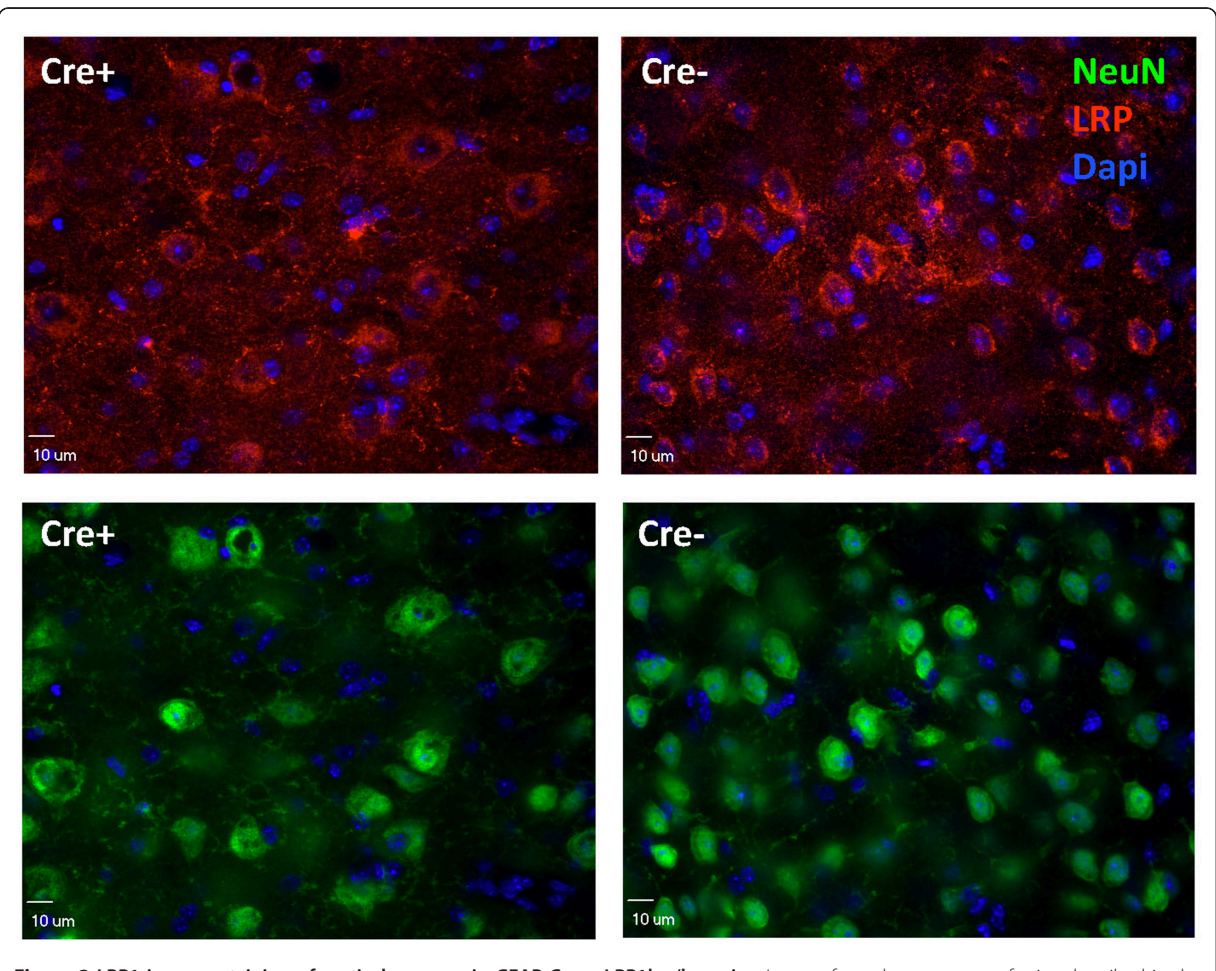
**LRP1 immunostaining of cortical neurons in GFAP-Cre × LRP1lox/lox mice**. Images from the same sets of mice described in the legend to Figure 2 were captured to show cortical levels of LRP1. LRP1 α377 antibody (1:1,000, red) was used to stain LRP1; NeuN (1:1,000, green) was used to stain neurons; Dapi in the mounting reagent was used to display nuclei. There was no obvious difference between mice transgenic for GFAP-Cre and those lacking GFAP-Cre.

In immunohistochemical stains of hippocampus of mice that were double transgenic for APPswe/PS1dE9 and GFAP-Cre in the LRP1lox/lox background, we observed very little staining for LRP1 in hippocampal neurons in the CA fields and dentate gyrus (DG) (Figure 2; Additional file 2 Figure S1). In contrast, in APPswe/PS1dE9/LRP1lox/lox mice we observed strong immunoreactivity for LRP1 in these regions (Figure 2). This pattern of reactivity matches what would be expected based on the levels of mRNA in these structures (Additional file 3 Figure S2). In the cortex of mice of the same genotype, we observed that most neurons, identified by NeuN staining, remained positive for LRP1 immunoreactivity, albeit at what appeared to be slightly lower levels, indicating that the recombination of the flox'ed LRP1 allele was less complete in cortical neurons (Figure 3). Thus, although the level of recombination in the cortex was less than reported for other flox'ed alleles [27], the level of recombination in the hippocampus was much more robust and thus we focused our analysis on amyloid deposition within the hippocampus.

**Figure 4:**
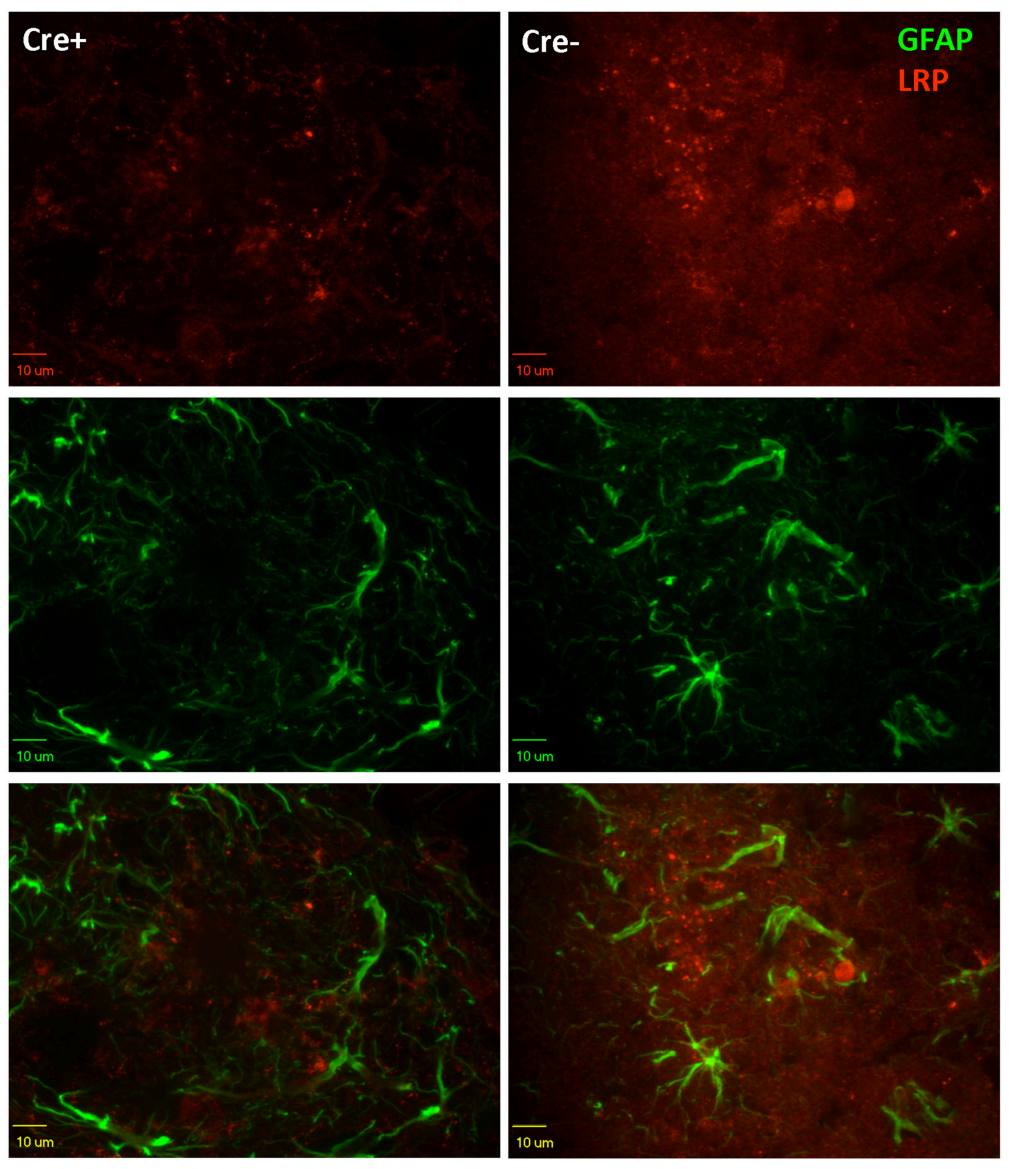
**LRP1 is below the level of detection in astrocytes**. The same sets of mice described in the legend to Figure 2 were immunostained. LRP1 α377 antibody (1:1,000, red) was used for LRP1 staining and antibody to GFAP (1:1,000, green) was used to mark astrocytes. Reactive astrocytes could be seen around amyloid plaques in APPswe/PS1dE9 × LRP1lox/lox (right column) but these cells did not show detectable levels of LRP1. Immunoreactivity to LRP1 was evident within the plaque as neuritic profiles. LRP1 immunostaining in these neuritic structures was diminished in most hippocampal amyloid plaques of APPswe/PS1dE9 × LRP1lox/lox × GFAP-Cre mice (left column).

Based on previous studies, we expected that LRP1 would be expressed at some level in astrocytes, particularly activated astrocytes [2-4]. No LRP1 immunoreactivity could be detected in resting astrocytes of any genotype (Additional file 4 Figure S3). In the hippocampus of nine-month-old GFAP-Cre/APPswe/PS1dE9/LRP1lox/lox mice, we observed that amyloid deposits still formed at a relatively high frequency (see below) and around these deposits we observed activated astrocytes. However, we failed to detect LRP1 in activated astrocytes surrounding these plaques (Figure 4). Within these deposits we observed LRP1 immunoreactivity that was reminiscent of neuritic profiles (Figure 4). These LRP1 immunoreactive neuritic profiles were mostly absent within the molecular layer of the hippocampus of APPswe/PS1dE9/LRP1lox/lox mice that were positive for GFAP-Cre.

Collectively, these data indicate that the levels of LRP1 are inherently low in astrocytes, including activated astrocytes, and that expression of Cre via the GFAP promoter produces efficient recombination of the LRP1 gene in neurons of the hippocampus. We presume that any expression of LRP1 in astrocytes was also reduced in LRP1lox/lox mice that were transgenic for GFAP-Cre but the absence of detectable staining in the absence of GFAP-Cre makes it impossible to confirm this assumption.

### Hippocampal deposition of Aβ is not altered by reduction in LRP1 levels

**Figure 5:**
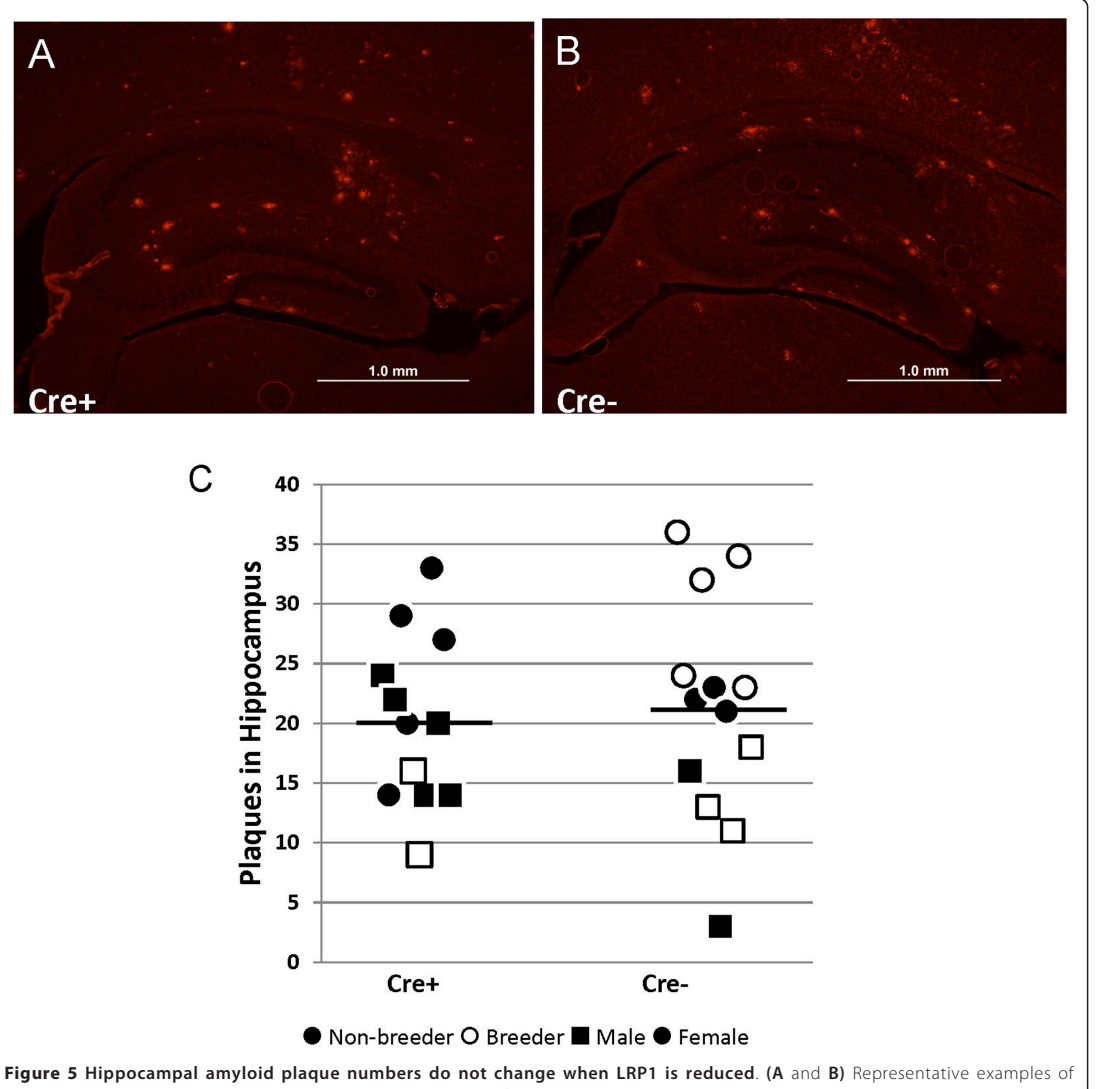
**Hippocampal amyloid plaque numbers do not change when LRP1 is reduced**. **(A **and **B) **Representative examples of immunohistochemical stains with 6E10 antibody (1:1,000, Covance) for Aβ in APPswe/PS1dE9/LRP1lox/lox mice that were either positive or negative for GFAP-Cre. **(C) **To quantify amyloid deposits, we stained sections with Thioflavin-S, which produces a sharper distinction between tissue and amyloid plaques (see Additional file 5 Figure S4 for an example) and captured images. The hippocampus was outlined and then the number of amyloid plaques inside the hippocampus was counted manually. A dot plot of amyloid plaque numbers in mice that were Cre+ is compared to the numbers in mice that were Cre-. Animals that were used as breeders are noted. No significant differences between genotypes either Cre+ (n = 12) vs. Cre- (n = 13) groups (*P *= 0.75) or between breeders (n = 10) vs. non-breeders (n = 15) (*P *= 0.69) was noted.

In the hippocampus of nine-month-old APPswe/PS1dE9/LRP1lox/lox mice, we observed no obvious difference in the number of Aβ immunoreactive deposits in mice that were positive for GFAP-Cre as compared to mice lacking GFAP-Cre (Figure 5). With the lack of an obvious difference in amyloid plaque burden in APPswe/PS1dE9 mice lacking LRP1 expression in the hippocampus, we sought to carefully quantify plaque numbers in the hippocampus of multiple sections at multiple levels. Tissue sections were stained with Thioflavin S as a means of detecting amyloid deposits and then the hippocampus was outlined and the number of deposits counted (see Additional file 5 Figure S4). The number of deposits in the hippocampus of APPswe/PS1dE9/LRP1loxp/loxp mice (n = 13) were similar to APPswe/PS1dE9/LRP1 loxp/loxp/GFAP-Cre mice (n = 12), meaning that significantly reducing LRP1 expression in the hippocampus had no obvious effect on the number or distribution of amyloid deposits in this structure. Female mice of all genotypes showed slightly higher levels of amyloid deposits as compared to males (Figure 5). We assessed whether loss of LRP1 altered any of the morphological features of the plaque such as density of neuritic structures, finding no obvious difference by silver staining (Additional file 6 Figure S5).

### Total Aβ levels do not change when LRP1 levels are reduced

**Figure 6:**
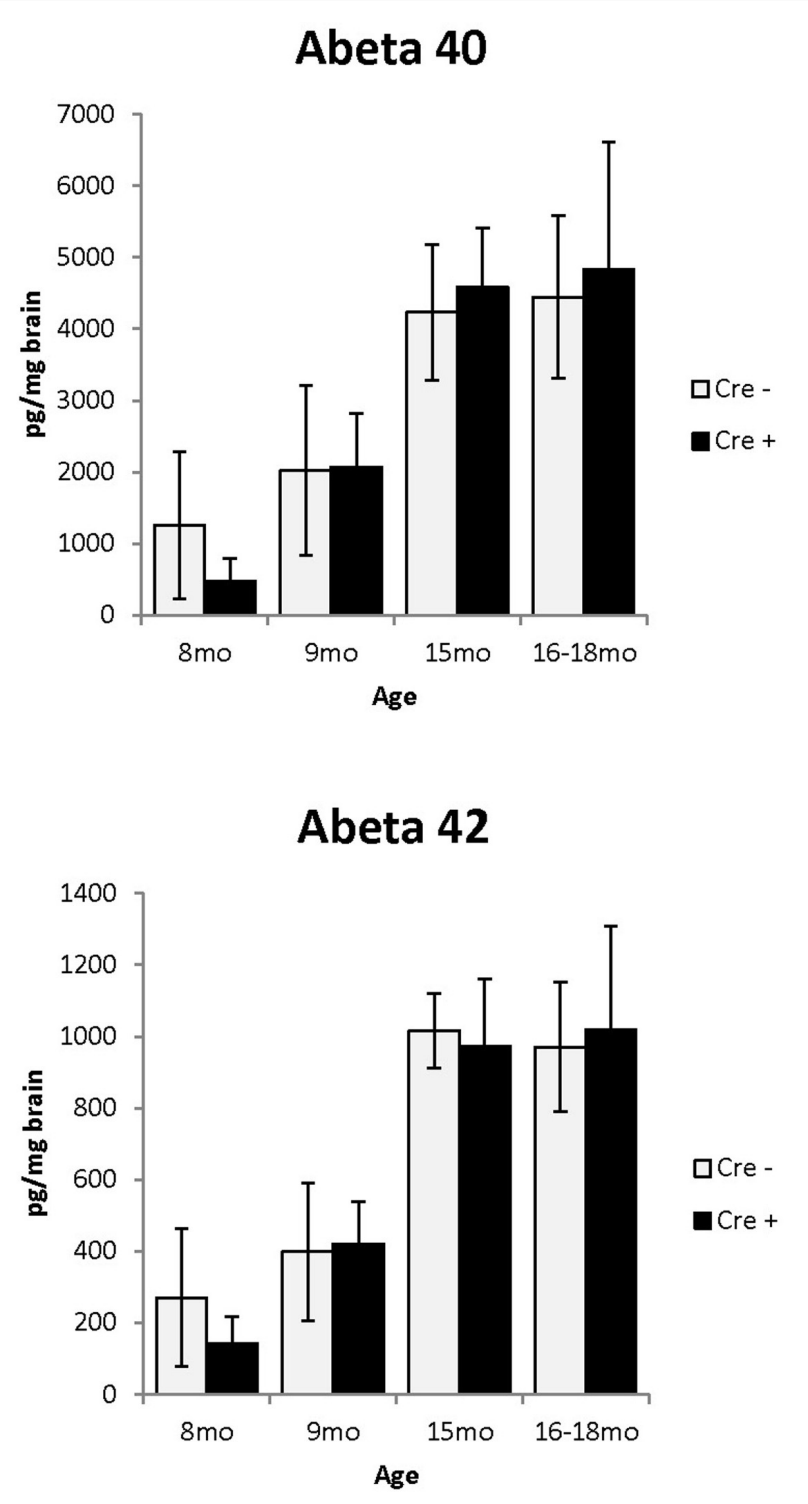
**Aβ ELISA assay of whole brain homogenates show no differences in Aβ levels by genotype**. Aβ levels in 8-, 9-, 15- and 16 to 18-month-old with normal LRP1 levels (APPswe/PS1dE9(+)/LRP1lox/lox) were compared to those with lower LRP1 levels (GFAP-Cre +). There was no difference in Aβ levels by genotype by age (p values range 0.40 to 0.90 based on Student's *t*-test). ELISA data used to generate the graph are shown in Table 1, which includes information on the number of animals used at each age.

Sandwich ELISAs were used to measure the total amount of Aβ40 and Aβ42 in the brains of these mice as another means to assess amyloid burden. To completely solubilize different forms of the peptides, aggregated or otherwise, homogenates were incubated for several hours in guanidine-HCl before being diluted in buffer for ELISA. Consistent with results from the amyloid plaque counting, ELISA measurements confirmed that the levels of Aβ40 and 42 in APPswe/PS1dE9 mice with reduced levels of LRP1 were no different from that of APPswe/PS1dE9 mice with normal levels of LRP1 (Table 1 and Figure 6). Further, a regression analysis to plot Aβ levels at different ages showed that the rate of Aβ accumulation did not change by the reduction of LRP1 levels (Additional file 7 Figure S6).

**Table 1:** The levels of Aβ in the brains of mice of each genotype and age

Age (month)	Cre	N	Aβ40 (pg/mg brain)			Aβ42 (pg/mg brain)			ratio 40/42
			Average	STDEV.P	*P*-value	Average	STDEV.P	*P*-value	
**8**	-	3	1,257	1,022	0.40	271	193	0.45	4.6
	+	3	481	315		142	75		3.4
**9**	-	17	2,024	1,192	0.90	399	192	0.71	5.1
	+	12	2,074	746		421	116		4.9
**15**	-	4	4,235	946	0.65	1,015	104	0.74	4.2
	+	4	4,582	823		972	188		4.7
**16 to 18**	-	4	4,445	1,135	0.74	971	181	0.79	4.6
	+	5	4,830	1,777		1,020	289		4.7

There was no difference in the levels of Aβ40 and 42 in the brains of mouse expressing APPswe/PS1dE9 gene in the LRP1 lox/lox homozygous background in the absence or presence of the GFAP-Cre transgene in any given age group.

## Discussion

In the present study, we have used genetic manipulation to reduce the expression levels of LRP1 in the hippocampus of mice that co-express mutant APP and mutant PS1. We demonstrate that lowering the levels of LRP1 by approximately 50% throughout the brain and possibly by a much greater degree in hippocampus had no significant impact on the rate, severity or character of amyloid deposition. We found no evidence of a proportional change in hippocampal amyloid burden by reducing LRP1 expression in CA and DG neurons with no obvious morphological change in the appearance of the plaques that formed.

To reduce LRP1 levels in the brains of APPswe/PS1dE9 mice, we employed mice that express Cre recombinase under the transcriptional regulation of the GFAP promoter and mice in which the LRP1 allele has been modified by introduction of lox p sites. The GFAP-Cre mice have previously been used in several studies and these investigations have previously demonstrated that Cre is expressed early in neurogenesis such that targeted genes may recombine in both neurons and glia [26,27]. In our cross of the GFAP-Cre mice with LRP1lox/lox mice, we observed efficient targeting of LRP1 expression in the hippocampal neurons. However, cortical neurons continued to show demonstrable LRP1 immunoreactivity. We note, that in the hippocampus, a subset of neurons in the molecular layer continued to express LRP1 in GFAP-Cre/loxp/loxp mice; however, the levels in CA and DG neurons were dramatically reduced. The levels of LRP1 in astrocytes appeared to be inherently low and we could not confirm whether or not expression of LRP1 was reduced further in LRP1lox/lox mice harboring the GFAP-Cre.

We observed no obvious developmental abnormality or reductions in the numbers of CA and DG neurons in mice transgenic for GFAP-Cre and LRP1 loxp/loxp, or mice of this genotype that were also transgenic for APPswe/PS1dE9. However, as noted in Results, we encountered significant difficulty in breeding these animals and noted a high degree of lethality early in the process. For reasons that remain unknown, these issues were overcome using a strategy of foster mothering with FVB/NJ females. The APPswe/PS1dE9 mice and the LRP1 loxp mice were both congenic in the C57BL/6J background and we bred the GFAP-Cre into C57BL/6J mice for four generations before beginning the crosses to produce experimental animals. In our hands, mice of the C57BL/6J strain are poorer mothers than commonly used hybrid strains or the inbred FVB/NJ strain. As we expected, foster mothering solved issues we had with poor mothering by C57BL/6J females. Unexpectedly, offspring that were foster-mothered by FVB/NJ females survived much better than mice of the same genotype mothered by C57BL/6J females. We presently have no explanation for this outcome. We note that in a previous effort to cross congenic C57BL/6J APPswe/PS1dE9 mice to congenic C57BL/6J RAP knockout mice we observed a high level of lethality in mice that were transgenic for APPswe/PS1dE9 and homozygous knockout for RAP [22]. RAP is a direct ER chaperone for LRP1 and loss of RAP lowers the level of mature LRP1 that reaches the cell surface [37]. Notably, others observed no issues in crosses of the PDAPP mouse model to RAP knockout mice [23], so we concluded that the lethality we observed our earlier work was due to some interaction between mutant PS1 and RAP. Our experience in the mating of the LRP1 loxp mice to APPswe/PS1dE9 mice leads us to believe that there may be some more complex interaction between PS1dE9 and RAP/LRP1 that reduces viability. This current study defines the source of this defect to the nervous system because LRP1 is only reduced in the brain by the expression of GFAP-Cre. Although likely to be unrelated to AD, further examination of the basis of this lethality may reveal functions of APPswe or PS1dE9 that impact CNS function.

Because the reduction in neuronal LRP1 was greatest in the hippocampus, we focused our pathologic analyses on the hippocampus, finding no significant change in the severity of amyloid deposition when LRP1 levels were reduced in the APPswe/PS1dE9 mice. The data shown in Figure 5C document the variability in the number of amyloid deposits in hippocampus between mice that are positive and negative for Cre recombinase. In both groups, the number of animals that were below and above the mean (horizontal line) was similar. Notably, if the number of deposits had been reduced by just 30% (with the same level of variance), then we would have been able to have reached statistical significance (*P *= 0.05) with just 10 animals in each group (our n's were 12 vs. 13). Based on quantification of immunoblots from hemi-brains, we conservatively estimate that the minimum reduction in LRP1 levels would be in the range of 50%. By power calculation, we should have been powered to observe a 30% decrease in amyloid plaque numbers and thus we conclude that reducing LRP1 levels does not produce a proportional decrease in amyloid plaque numbers. Consistent with our findings, Zerbinatti and colleagues reported that increasing the levels of LRP1, via expression of a recombinant mini-receptor, did not produce a proportional increase in amyloid burden [25]. However, this study did note small increases of levels of both soluble and insoluble Aβ in the cortex with a more selective increase in soluble Aβ in the hippocampus of older mice with high amyloid burden. Our measures of total Aβ did not find evidence that reducing LRP1 levels produced a change in the accumulated levels of Aβ. Zerbinatti and colleagues also noted the appearance of Aβ42 dimers after extraction in buffers containing 0.1 M carbonate. We have not been able to reliably detect dimeric Aβ in our APPswe/PS1dE9 mice at any age and thus we cannot comment as to whether reducing LRP1 reduces the levels of these dimers.

Interpreting the outcome of this work is not as straightforward as one would like because the LRP1 levels were not reduced to zero. One issue is that we do not know how much LRP1 must be expressed to sustain amyloidogenesis or whether we should expect changes in amyloid formation in proportion to changes in LRP1 levels. Additionally, the source of the Aβ peptides that produce deposits may be neurons that project axons into the hippocampus as well as local neurons. Within the hippocampus, we observed a near elimination of LRP1 immunoreactivity of neurons of the CA fields. However, the hippocampus receives axonal projections from the entorhinal cortex making it possible that an activity of LRP1 in cortical neuronal projections masked any effect of local reductions in LRP1 levels in hippocampus. Ultimately, the most conservative interpretation is that significant suppression of LRP1 activity in local hippocampal neurons does not produce a proportional decrease in amyloid plaque numbers this structure.

Apart from the potential effects of LRP1 reductions on amyloid plaque levels, we had anticipated that we might see some change in neuritic pathology. Nearly all amyloid plaques in APPswe/PS1dE9 mice contain apolipoprotein E (ApoE) [31] and ApoE is known to be a ligand for LRP1 that modulates neurite outgrowth [38]. Thus, we had thought it possible that reducing LRP1 locally in the hippocampus could have reduced neuritic pathology around amyloid plaques. Notably, in APPswe/PS1dE9/LRP1lox/lox mice that were positive for GFAP-Cre we observed large neuritic plaques in which the neurites were depleted of LRP1 immunoreactivity.

## Conclusions

This study used genetic tools to reduce LRP1 levels in the brains of mice that co-express APPswe/PS1dE9 and develop amyloid pathology, producing a model in which LRP1 levels in most hippocampal neurons were lowered to below the level of detection by immunohistochemistry. In the hippocampus of mice with reduced LRP1 levels, we observed no obvious change in the rate of deposition, severity, or character of amyloid deposits. Obviously, this study does not provide any insight into what LRP1 expressed in non-neural cell types, such as endothelial cells, other organs may do in regard to modulating amyloid deposition. It is also very possible that homologues of LRP1 may have compensated for any effect of reducing the levels of LRP1. However, at minimum, our study indicates that reducing the levels of LRP1 does not produce proportional reductions in amyloid plaque numbers in the hippocampus of the APPswe/PS1dE9 model. Thus, this genetic test does not produce supportive evidence for the notion that modulating LRP1 function in neurons or glial could be beneficial in modulating amyloidosis. Whether this outcome is unique to the APPswe/PS1dE9 model is of course an issue in assessing the potential importance of this pathway in human disease.

## Abbreviations

ApoE: apolipoprotien E; APP: amyloid precursor protein; APPswe/PS1dE9: a tandem integration of transgenes encoding amyloid precursor protein with the Swedish mutation and presenilin 1 gene with exon 9 deletion; BCA: bicinchoninic acid assay: a biochemical assay for protein concentration; CA: cornu ammonis in hippocampus; Cre-lox: Cre recombinase-Lox P site system, a genetic tool to control site specific recombination events in genomic DNA; DG: dentate gyrus in hippocampus; ELISA: enzyme-linked immunosorbent assay; GFAP-Cre: transgenic mice with Cre recombinase driven by glial fibrillary acidic protein (GFAP) promoter; LRP1: low-density lipoprotein receptor-related protein.

## Competing interests

The authors declare that they have no competing interests.

## Authors' contributions

All authors contributed to this manuscript. GX designed the experiments, participated to generate the cohorts of mice, carried out most of the experiments, performed the statistical analysis, and drafted the manuscript. CG contributed to the histology, immunostaining work and collected data of amyloid plaques. SF genotyped the mice and recorded the breeding data. DRB conceived of the whole study, participated in detailed experimental design and prepared the manuscript. All authors read and approved the final manuscript.

## Supplementary Material

Additional file 1 **Genotype distribution of the transgenic mice used in this study**. In total, 325 transgenic mice with LRP1 lox/lox background were generated. The mice listed in the table are those that reached weaning age and survived to at least 1.5 months of age. The actual and expected percentages of mice of each genotype are listed. The predicated frequency rate is based on Mendelian distribution. As is typical for APPswe/PS1dE9 mice that are found dead, there were no obvious birth defects to account for the early lethality (Table S1). Click here for file

Additional file 2 **Low power image of LRP1 immunostaining in hippocampus of APPswe/PS1dE9/LRP1 lox/lox mice that are positive or negative for GFAP-Cre**. Free-floating frozen sections were immunostained as described in Methods of the main text. The images shown are representative of what was observed in at least three animals of each genotype (Figure S1). Click here for file

Additional file 3 **Low power image captured from the Allen Brain Atlas**. The image shown here was captured from the website for the Allen Brain Atlas for the adult mouse brain. The gene name searched for was LRP1. The view shown displays false color for expression levels of a sagittal view (Figure S2). Click here for file

Additional file 4 **Resting astrocytes are not immunoreactive to LRP1 antibodies**. The images shown are high power images of hippocampus from APPswe/PS1dE9/LRP1 lox/lox mice that were either positive or negative for GFAP-Cre. We observed no obvious reactivity in cells showing a morphology identified by GFAP staining and there was no indication of significant co-localization of GFAP and LRP1 immunoreactivity. The images shown are representative of what was observed in at least three animals of each genotype (Figure S3). Click here for file

Additional file 5 **Thioflavin-S staining of hippocampal sections**. The numbers of amyloid plaques in the hippocampus as identified by Thioflavin-S staining section were manually by outlining the border of the hippocampus on the image and then counting the number of plaques in hippocampus. These images show representative examples of animals of both genotypes (Figure S4). Click here for file

Additional file 6 **The morphology of hippocampal amyloid deposits is unchanged by the lack of LRP1**. Silver stains of 30 μm sections from APPswe/PS1dE9 × LRP1 lox/lox mice that were either positive or negative for GFAP-Cre. White arrows mark neurites and the white arrow heads mark to the core of the plaques (Figure S5). Click here for file

Additional file 7 **Regression analysis of Aβ levels in mice of each genotype by age**. No difference in the rate of Aβ accumulation was evident. The regression curve of the slopes between two groups of animals is not significant for Aβ40 (*P *= 0.409) or Aβ42 (*P *= 0.864) (Figure S6). Click here for file
