# Supplementary material for: Reduction of low-density lipoprotein receptor-related protein (LRP1) in hippocampal neurons does not proportionately reduce, or otherwise alter, amyloid deposition in APPswe/PS1dE9 transgenic mice
Source: Alzheimers Res Ther. 2012 Apr 26;4(2):12. doi: 10.1186/alzrt110 (PMC4054673; doi:10.1186/alzrt110)
Supplement: Additional file 1 — Genotype distribution of the transgenic mice used in this study. In total, 325 transgenic mice with LRP1 lox/lox background were generated. The mice listed in the table are those that reached weaning age and survived to at least 1.5 months of age. The actual and expected percentages of mice of each genotype are listed. The predicated frequency rate is based on Mendelian distribution. As is typical for APPswe/PS1dE9 mice that are found dead, there were no obvious birth defects to account for the early lethality (Table S1). [file alzrt110-S1.DOCX]

| **Genotypes** | **APP/PS1** | **+** | **-** | **+** | **-** | **Total** |
| --- | --- | --- | --- | --- | --- | --- |
|  | **GFAP-Cre** | **-** | **+** | **+** | **-** |  |
|  | **LRP** | **Lox/lox** | **Lox/lox** | **Lox/lox** | **Lox/lox** |  |
| **Expected frequency of offspring** | | 25% | 25% | 25% | 25% | 100% |
| **Non-**  **fostered** | Number of mice identified by genotype | 56 | 31 | 21 | 50 | 158 |
|  | Observed frequency | 35% | 20% | 13% | 32% | 100% |
|  | Estimated number of mice that died before 1.5 months of age (age of genotyping) | - | ~20 | ~30 | - | ~50 |
|  | Number of mice found dead (< 9mos old) | 2 | 3 | 9 | 1 | 15 |
|  | (Premature death rate in group after weaning) | (4%) | (10%) | **(43%)** | (2%) | (9%) |
|  | Age range of mice found dead (months) | (2.3-6.3) | (1.9-8.8) | (2.4-4.6) | 3.3 |  |
| **Fostered** | Number of Mice | 45 | 41 | 32 | 49 | 167 |
|  | Observed frequency | 27% | 25% | 19% | 29% | 100% |
|  | Estimated number of mice that died before 1.5 months of age (age of genotyping) | - | ~5 | ~15 | - | ~20 |
|  | Number of mice found dead (< 9mos old) | 4 | 2 | 1 | 0 | 7 |
|  | (Premature death rate in group after weaned) | (9%) | (5%) | (3%) | (0%) | (4%) |
|  | Age range of mice found dead (months) | (2.0-9.0) | (2.8-7.7) | 3.7 | - |  |
