# Supplementary figures and images for: Reduction of low-density lipoprotein receptor-related protein (LRP1) in hippocampal neurons does not proportionately reduce, or otherwise alter, amyloid deposition in APPswe/PS1dE9 transgenic mice
Source: Alzheimers Res Ther. 2012 Apr 26;4(2):12. doi: 10.1186/alzrt110 (PMC4054673; doi:10.1186/alzrt110)

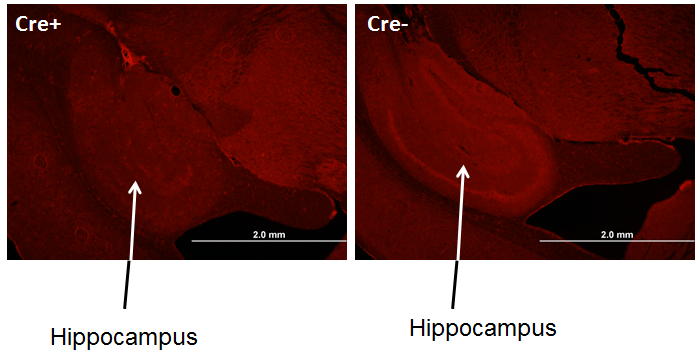

Supplement: Additional file 2 — Low power image of LRP1 immunostaining in hippocampus of APPswe/PS1dE9/LRP1 lox/lox mice that are positive or negative for GFAP-Cre. Free-floating frozen sections were immunostained as described in Methods of the main text. The images shown are representative of what was observed in at least three animals of each genotype (Figure S1). [file alzrt110-S2.TIFF]

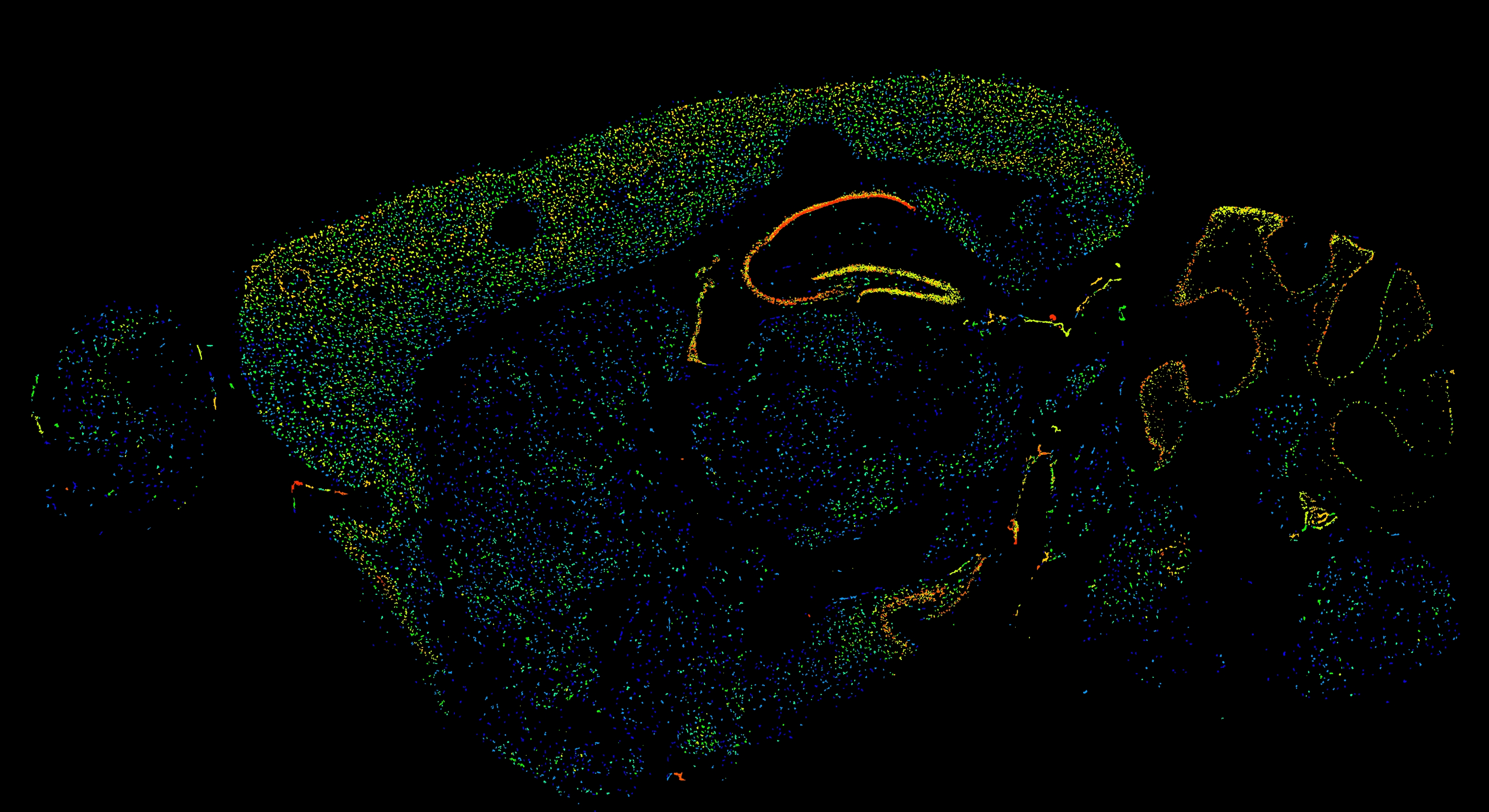

Supplement: Additional file 3 — Low power image captured from the Allen Brain Atlas. The image shown here was captured from the website for the Allen Brain Atlas for the adult mouse brain. The gene name searched for was LRP1. The view shown displays false color for expression levels of a sagittal view (Figure S2). [file alzrt110-S3.JPEG]

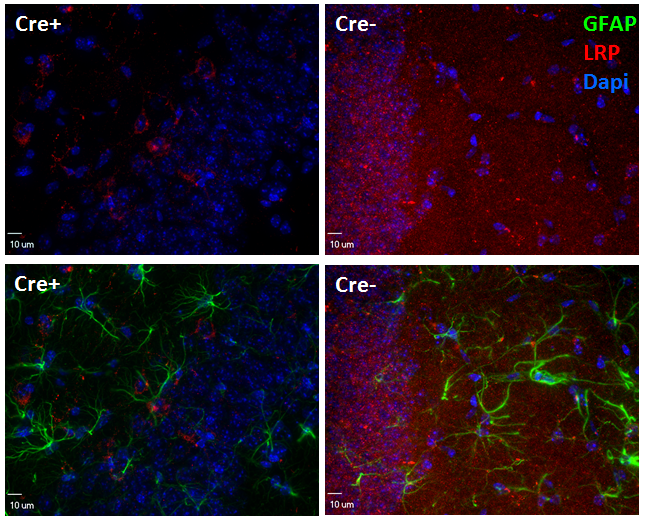

Supplement: Additional file 4 — Resting astrocytes are not immunoreactive to LRP1 antibodies. The images shown are high power images of hippocampus from APPswe/PS1dE9/LRP1 lox/lox mice that were either positive or negative for GFAP-Cre. We observed no obvious reactivity in cells showing a morphology identified by GFAP staining and there was no indication of significant co-localization of GFAP and LRP1 immunoreactivity. The images shown are representative of what was observed in at least three animals of each genotype (Figure S3). [file alzrt110-S4.TIFF]

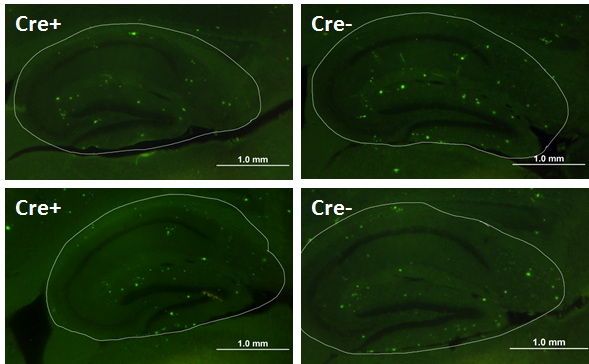

Supplement: Additional file 5 — Thioflavin-S staining of hippocampal sections. The numbers of amyloid plaques in the hippocampus as identified by Thioflavin-S staining section were manually by outlining the border of the hippocampus on the image and then counting the number of plaques in hippocampus. These images show representative examples of animals of both genotypes (Figure S4). [file alzrt110-S5.TIFF]

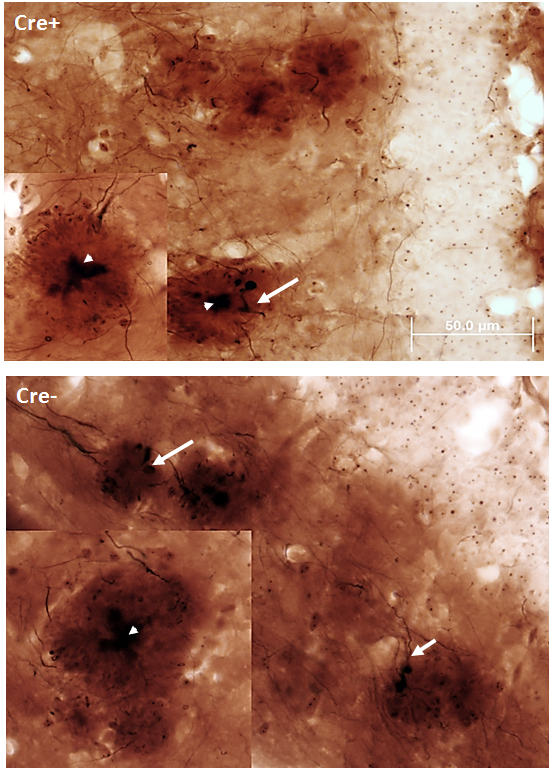

Supplement: Additional file 6 — The morphology of hippocampal amyloid deposits is unchanged by the lack of LRP1. Silver stains of 30 μm sections from APPswe/PS1dE9 × LRP1 lox/lox mice that were either positive or negative for GFAP-Cre. White arrows mark neurites and the white arrow heads mark to the core of the plaques (Figure S5). [file alzrt110-S6.TIFF]

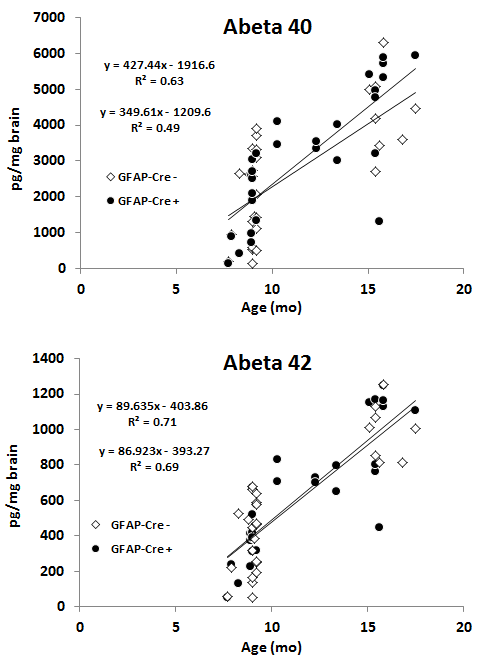

Supplement: Additional file 7 — Regression analysis of Aβ levels in mice of each genotype by age. No difference in the rate of Aβ accumulation was evident. The regression curve of the slopes between two groups of animals is not significant for Aβ40 (P = 0.409) or Aβ42 (P = 0.864) (Figure S6). [file alzrt110-S7.TIFF]
